# Rapamycin inhibition of baculovirus recombinant (BVr) ribosomal protein S6 kinase (S6K1) is mediated by an event other than phosphorylation

**DOI:** 10.1186/1478-811X-10-4

**Published:** 2012-03-01

**Authors:** Mushtaq A Beigh, Mehvish Showkat, Mahboob ul Hussain, Shafat A Latoo, Sheikh T Majeed, Khurshid I Andrabi

**Affiliations:** 1 Department of Biotechnology, Science block, University of Kashmir, Jammu and Kashmir 190006, India

**Keywords:** S6 kinase, Baculovirus, Rapamycin, mTOR, Threonine 412 and Threonine 252

## Abstract

**Background:**

Ribosomal protein S6 kinase 1(S6K1) is an evolutionary conserved kinase that is activated in response to growth factors and viral stimuli to influence cellular growth and proliferation. This downstream effector of target of rapamycin (TOR) signaling cascade is known to be directly activated by TOR- kinase mediated hydrophobic motif (HM) phosphorylation at Threonine 412 (T412). Selective loss of this phosphorylation by inactivation of TOR kinase or activation/recruitment of a phosphatase has accordingly been implicated in mediating inhibition by rapamycin.

**Findings:**

We present evidence that baculovirus driven expression of S6K1 in insect cells (Sf9) fails to activate the enzyme and instead renders it modestly active representing 4-6 folds less activity than its fully active mammalian counterpart. Contrary to the contention that viral infection activates TOR signaling pathway, we report that BVr enzyme fails to exhibit putative TOR dependent phosphorylation at the HM and the resultant phosphorylation at the activation loop (AL) of the enzyme, correlating with the level of activity observed. Surprisingly, the BVr enzyme continued to exhibit sensitivity to rapamycin that remained unaffected by mutations compromised for TOR phosphorylation (T412A) or deletions compromised for TOR binding (ΔNH _2-46_/ΔCT_104_).

**Conclusions:**

These data together with the ability of the BVr enzyme to resist inactivation by phosphatases indicate that inhibition by rapamycin is not mediated by any phosphorylation event in general and TOR dependent phosphorylation in particular.

## Findings

S6K1 is a ubiquitously expressed serine/threonine protein kinase that phosphorylates 40S ribosomal protein S6, and coordinates cellular growth and proliferation. Multiple independent phosphorylations have been proposed to account for complete activation of the enzyme in response to growth factor stimulation [1]. A battery of protein kinases coordinate to accomplish the activation of the enzyme through a series of phosphorylation events that culminate in phosphorylating Threonine 412 (T412) at HM and Threonine 252 (T252) at the AL [2-7]. The dynamics of these critical phosphorylations, in particular the one at the HM therefore, dictates the activation state of the enzyme. Accordingly selective loss of this TOR kinase dependent phosphorylation has been implicated in mediating the inhibitory effects of rapamycin, through direct inactivation of TOR kinase, or through activation/recruitment of a phosphatase [8-11]. In addition to Insulin and other growth factor stimulation, S6K1 has also been reported to get activated in response to viral infection, such that baculovirus mediated expression of the enzyme in insect cells activates the enzyme by phosphorylation at similar sites as identified in the enzyme from regulated cells [12]. Since it stands established that insect S6 kinase, behaves similar to that of its mammalian counterpart, it is conceivable that the activation state of the enzyme and its inhibition by rapamycin would be no different than the one established for mammalian systems [13,14]. Furthermore, since the stimulus due to viral infection can at no point be disengaged in Sf9 cells, the state of S6K1 activation could be deemed as constitutive and therefore, ideal to investigate the dynamics of activating phosphorylations in presence of rapamycin. Herein we provide evidence that activity or rapamycin sensitivity of Baculovirus recombinant enzyme (BVr) is not dependent on any posttranslational phosphorylation events in general and TOR- mediated phosphorylation in particular.

**Figure 1:**
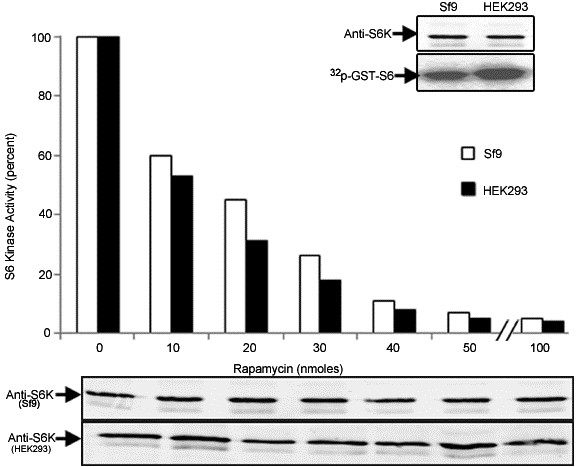
**Comparative activity and rapamycin response of BVr and HEK S6K**. Sf9 cells infected with HA-S6K1α1 virus at an M.O.I of ≤ 1 in TNM-FH media supplemented with 5% FBS and HEK-293 cells transiently transfected with HA-S6K1 in DMEM containing 5% FBS were grown for 24 and 48 hours respectively. Cells were exposed to rapamycin or ethanol for 15 min at indicated concentrations before harvest. Lysates were clarified and used for inmunoprecipitation with α HA antibody. Immune complexes recovered were subjected to kinase assays in presence of ^32^pATP using GST-S6 or S6 peptide as a substrate. Samples were separated on a 12% SDS-PAGE gel transferred on to a PVDF membrane for autoradiography or immunoblotting. Peptide kinase assays were performed by standard procedure and the activity in Cpm (Counts per minute) normalized to 100% for comparison. The data is a representative expression of four independent experiments.

**Figure 2:**
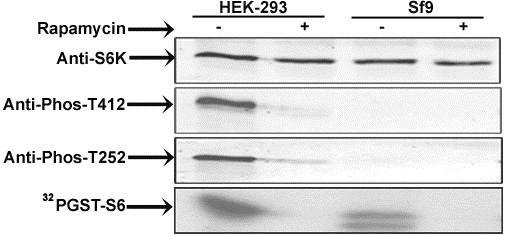
**Status of activating phosphorylations in HEK and BVrS6K1**. HA-S6Kα1 immuno-precipitated from HEK 293 cells or Sf9 cells treated with or without rapamycin (50nM), were subjected to immune-complex kinase assay using GST-S6 as substrate and separated on 12% SDS-PAGE gel for autoradiography. Alternatively immune complexes were transferred on to a PVDF membrane for probing with indicated antibodies. The results represent typical of three independent observations.

**Figure 3:**
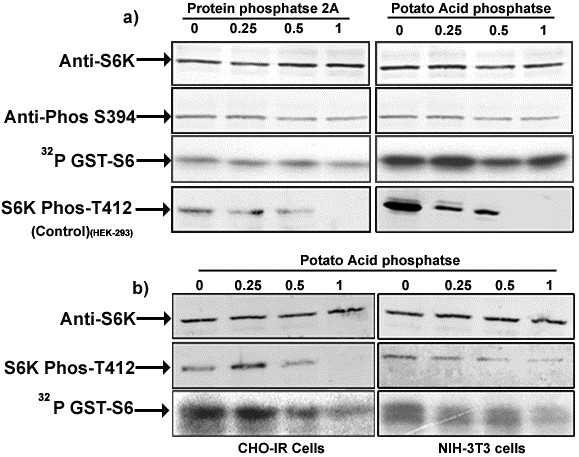
**BVr-S6K1 is resistant to phosphatase inactivation**. S6Kα1-WT immuno-precipitated with anti-HA antibodies from infected Sf9, HEK 293, CHO or NIH-3T3 cells as above. The immunoprecipitate was split into aliquots and incubated with indicated concentrations of acid phosphatase (3a, 3b) or protein phosphatase 2A (b) for 30 min and washed with buffer containing phosphatase inhibitors or okadaic acid (5nM) respectively, followed by excessive washing with kinase buffer and processed for S6 phosphorylation assay as above.

**Figure 4:**
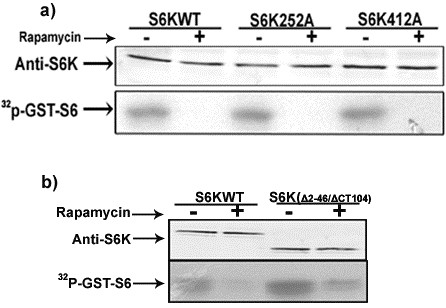
**Tor signaling input is not required for rapamycin inhibition of BVr enzyme**. Sf9 cells were infected with S6Kα1WT, S6Kα1T412A and T252A (a) or S6Kα1WT & S6K ΔNH_2-46_/ΔCT_104 _(b) recombinant viruses at an M.O.I ≤ 1. Cells were exposed to rapamycin (50 nM) or vehicle, 24 h post infection for 15 min and harvested, lysed for IPs and subjected to S6 kinase as above. The figure is representative of three independent experiments.

Baculovirus recombinant (BVr) ribosomal protein S6 kinase (S6K1), expressed in Sf9 cells described along with other methodological details in Additional file 1 was active towards phosphorylating GST-S6 in conformity with earlier findings [12]. The activity of the recombinant enzyme was 2-3 folds less than the random activity (without serum withdrawal or stimulation) exhibited by the enzyme transiently expressed in HEK 293 cells (Figure 1). Since the HEK enzyme could be activated a further 2-3 fold following stimulation, the BVr- enzyme in effect, was 4-6 fold less active than its mammalian counterpart. As seen in Figure 1, the BVr- enzyme was as sensitive to inhibition by rapamycin in a manner more or less comparable to the inhibition curve obtained for transiently expressed enzyme in HEK 293 cells. We have consistently observed slight recalcitrance of the enzyme to rapamycin inhibition, when the drug treatment is carried out without serum deprivation or after serum stimulation of the enzyme. Since serum or amino acid deprivation does not recreate the serum starved state in Sf9 cells, the concentration of rapamycin required to bring about inhibition was obviously higher than required otherwise. Furthermore the quantum of protein expression in Sf9 system was also an important determinant in establishing inhibitory concentration of the drug (not shown). Accordingly 20-24 hr post infection period, with a multiplicity of infection (MOI) of ≤ 1 was chosen as optimal time point where the level of recombinant protein was appropriate to achieve > 90% inhibition in activity at a concentration of 50 nM rapamycin. Immunoblot analysis using anti-phospho T412 and T252 antibodies easily established these phosphorylations in the enzyme immuno precipitated from HEK 293 cells, whose levels were seen to decrease considerably in the enzyme recovered from rapamycin treated cells (Figure 2). Surprisingly the antibodies failed to identify any of these phosphorylations in the BVr- enzyme in a series of experiments, even when the membranes with commasie stained bands were probed. The absence of these phosphorylations in the BVr- enzyme though conceivable in view of its lesser activity was surprising to account for its continued inhibition by rapamycin in the context of substantial evidence implicating these phosphorylations, especially T412 to mediate the inhibitory effects of the drug [11,15]. It could however, be argued that the presence of only a minute fraction of phospho T412 and T252 in the BVr- enzyme might escape detection through immuno blotting. That being the case, the BVr- enzyme would tend to be more sensitive to inactivation by phosphatase than otherwise. Figure 3 shows that potato acid phosphatase or phosphatase 2A failed to bring about any significant inactivation of the enzyme at concentrations that were effective in de-phosphorylating T412 from the HEK-293, CHO and NIH-3T3 immunoprecipitated enzyme, thereby disregarding the argument about the possible existence of a minor fraction of phospho T412 and T252 in the BVr enzyme. It is pertinent to emphasize that only a few important phosphorylation sites that include T252, T412 and Ser394 (S394) remain critical for activity in backdrop of the data that loss of phosphorylation sites in the carboxy terminal auto inhibitory domain (AID) does not bring about any appreciable change in the activity of the enzyme [6]. As such the resistance of the enzyme to phosphatase inactivation could only be explained if these sites were either absent or not accessible for phosphatase action. Since T412 and T252 are established post translational events, and the kinases that phosphorylate these sites identified [11,16,17] the contention of their inaccessibility was certainly not plausible. The only other site that assumed significance in terms of its requirements for enzyme activity in this system, S394 believed to be co-translational [18], understandably continued to resist phosphatase action (Figure 3a). Interestingly significant residual activity continues to be detected in the enzyme expressed in CHO-IR and NIH-3T3 cells even after the phosphorylation at T412 was more or less completely removed by phosphatase treatment (Figure 3b) lending credence to the observed resistance of BVr enzyme to phosphatase inactivation. We finally resorted to introduce phospho deficient mutations at the HM (T412) to Alanine (T412A) and AL (T252) to Alanine (T252A) to provide unequivocal evidence about their potential relevance or otherwise in influencing activity and or rapamycin sensitivity of the recombinant enzyme. Since neither mutation engendered any dramatic effects on either property of the enzyme (Figure 4a) it could be concluded that the activity of the BVr enzyme was not in any way due to either of the activating phosphorylations and neither phosphorylation was responsible for mediating the inhibitory effects of rapamycin. Since the activation and rapamycin sensitivity of the enzyme has also been shown to critically depend on the recruitment of TOR kinase through amino and carboxy terminal TOR signaling (TOS) motifs [19-21] it was imperative to examine, whether deletion of these motifs did indeed reproduce effects in accordance with the prevalent interpretations for mammalian cell system. Surprisingly the double mutant exhibited 2-3 fold more activity and partial resistance to rapamycin, in conformity with its reported behavior in mammalian cells (Figure 4b). However, the explanation attributing this mutation to facilitate direct phosphorylation at the HM is completely redundant in view of its absence in the BVr enzyme. It is therefore, safe to conclude that TOR recruitment and the resultant phosphorylation at the HM does not mediate the inhibitory effects of rapamycin. Increased activity associated with the mutant may therefore, be simply due to steric freedom achieved by the truncated version of the enzyme ordinarily accomplished by activating phosphorylations. Rapamycin may instead potentiate a cellular event other than phosphorylation via binding or dissociation of a regulatory factor that changes the conformation of the enzyme such that it fails to engender these critical phosphorylations. Accordingly truncations that override such constraints would activate the enzyme and override rapamycin inhibition, without necessary dependence on phosphorylations, as stands reported for other such truncations [22].

In the absence of the activating phosphorylations and lesser activity associated with the BVr-enzyme, it can be concluded that the viral infection *per se *does not activate the enzyme but instead appears to lock it in a state of activity comparable to a rather amplified basal (serum starved) state of the mammalian cells, in complete disagreement with the conclusion drawn by some investigators [12]. The stimulus required to bring about activating phosphorylations may therefore, be either absent in insect cells or inactivated due to viral infection, undermining the existence of kinases responsible to bring about both AL and HM phosphorylations [13,23]. It is therefore, obvious to contemplate that TOR signaling pathway that supposedly mediates both activation and rapamycin inhibition otherwise established in the insect cell system [13,24], fails to phosphorylate and activate the enzyme yet continues to mediate inhibition by rapamycin. Therefore, it seems that while the process of activation remains unaccomplished, viral infection preserves the events responsible for mediating rapamycin inhibition. In other words the activation and sensitivity to rapamycin appear to be two independent events, in complete contravention to prevalent hypotheses. Baculoviral infection in a way serves to provide a better system where the basal state, i.e. the form of the enzyme without phosphorylations at the HM and AL, is completely disengaged from the activation state otherwise quite difficult to achieve in a mammalian system. Since, it would be strange to contemplate a unique mechanism of rapamycin action for BVr-enzyme it is possible to contemplate the loss of activating phosphorylations in mammalian cells otherwise construed as mechanistic may in effect be only the consequence of rapamycin inhibition. It is however, imperative to validate the contention for better understanding of the process.

## Competing interests

The authors declare that they have no competing interests.

## Authors' contributions

MAB: Mutagenesis, production of recombinant viruses, viral titrations, Activity analysis of various recombinant proteins. MS: Expression of recombinant proteins in HEK 293 cells, Sf9 cell culture and expression. MUH: Technical supervision and data analysis. SAL: Culture and maintenance of HEK 293 cells, Standardization of protocols for transient transfection. STM: Cloning and sub cloning of some mutants in mammalian and baculoviral expression vectors. KIA: Design, overall supervision and preparation of the manuscript. All authors read and approved the final manuscript.

## Supplementary Material

Additional file 1 **The file contains detailed description of methods used in the communication**. Click here for file
