# Supplementary material for: Rapamycin inhibition of baculovirus recombinant (BVr) ribosomal protein S6 kinase (S6K1) is mediated by an event other than phosphorylation
Source: Cell Commun Signal. 2012 Mar 1;10:4. doi: 10.1186/1478-811X-10-4 (PMC3311567; doi:10.1186/1478-811X-10-4)
Supplement: Additional file 1 — The file contains detailed description of methods used in the communication. [file 1478-811X-10-4-S1.DOC]

**Materials and Methods**

**Materials and Antibodies**: Rapamycin and Protein G-Agarose beads (Calbiochem, USA), Potato acid phosphatase, Protein phosphatase 2A, Okadaic acid, DMEM, FBS, TNM-FH media, antibiotics were purchased from Sigma-Aldrich (St. Louis, MO) all other reagents were purchased from Sigma unless indicated, cellulose phosphate paper was from Whatman (Hillsboro, OR), γ-^32^pATP(10 mCi/ml) was purchased from BRIT( Mumbai ,India) ,Anti-S6K (Santa Cruz Biotechnologies Inc, USA), Anti-S6K Thr412 and Anti-S6K Ser394 were obtained from Cell Signaling technologies (Beverly MA), Anti-S6K Thr252 was from R&D systems (Minneapolis, MN), Anti-HA (Ascites HA-7 Clone Sigma), S6 peptide (Signal chem., Canada) PVDF membrane ( Millipore Inc Billerica, MA ), Goat Anti-Rabbit secondary antibody conjugated to IR Dye 800CW (LI-COR Biotechnology ,Lincoln, Nebraska).

| Set 1  (5'--3') | a | GTCACGCACGCATTTTGTGGAACAATAGAATACATGGCCCCTG |
| --- | --- | --- |
|  | b | CCACAAAATGCGTGCGTGACTGTTCCATCATGAATAGATTC |
| Set 2  (5'--3') | a | CTGGGTTTTGCATATGTGGCTCCATCTGTACTTGAAAGTGTG |
|  | b | GAGCCACATATGCAAAACCCAGAAAGACCTGGTTGG CACTTTCACTG |

**Plasmid constructs**: Influenza virus N-terminal Hemagglutinin(HA) epitope tagged Rattus S6 Kinase α1 (p85) (S6K1) and S6K Δ2-46 ΔCT104 cloned at EcoRI restriction site in pMT2 vector was a kind gift from Joseph Avruch (MGH, Harvard medical school). S6K T252A (Threonine replaced with Alanine) using primer Set1, S6K T412 A (Threonine replaced with Alanine) using primer Set2 by Quick changeTm site directed mutagenesis kit (Santa Clara, CA) by a protocol described in Zhang et al .

**Generation of GST-S6 (substrate):** 210 Bp sequence coding for C-terminal 69 aminoacids of S6 protein was PCR amplified using primer Set 4 and cloned in EcorI-XhoI site of pGEX-4T2 using S6 cDNA cloned in pDNR-Dual (Harvard plasmid database Clone ID: hs CD 00001811), as template and expressed in bacteria (BL21-DE3 p Lys S) and purified on GSH-Agarose beads (Sigma) and eluted using glutathione (Sigma) buffer and dialyzed against kinase buffer.

Generation of recombinant viruses: Recombinant viruses were generated by ligating EcoRI digested fragment from pMT2-S6KWT and various mutants in pVL-1392 and sequenced to check orientation and then co-transfected with linearized Baculo-viral DNA using Baculo-gold kit (BD Biosciences ,San Diego, CA) according to manufacturer’s instructions. Plaque assay was carried out to calculate viral titre strictly in accordance with manufacturer’s instructions.

**Cell culture and Transfections**: HEK293 cells were cultured in Dulbecco’s modified Eagle’s medium (with appropriate antibiotics) containing 10% (v/v) fetal bovine serum in an atmosphere of 5% Co2. Transfections were carried out using lipofectamine reagent (Invitrogen Carlsbad, California) as per manufacturer’s instructions. Insect cells (Sf9) were cultured in serum free TNM-FH media and seeded at cell density of 2x10^6^ cells per 60mm tissue culture plate and transfected with individual viruses for each of the above mentioned constructs with Multiplicity of Infection (M.O.I.) of ≤1 for 58-60 hours.

**Immuno-precipitations and Western blotting**: HEK-293 cells were starved overnight in DMEM containing 2% serum. HEK-293 and Sf9 cells were exposed to rapamycin (50ng/ml) for 20 minutes before harvest. Cells were lysed on ice in a buffer containing 50mM Tris-Cl (pH 7.5), 10mM MgCl2, 5mM EDTA, 2mM DTT, 50mM β-Glycero-phosphate, 0.5% Triton X-100 and Protease inhibitor cocktail (Sigma) for 30 minutes and centrifuged at 15000g for 30 minutes to clear the lysate. Cleared lysate was incubated with Anti-HA antibody immobilized on protein G- Agarose beads overnight. Beads were washed thrice with lysis buffer containing 500mM NaCl and a final wash with kinase buffer to remove the salt.

**Immune complex kinase assays**: S6Kα1WT and its mutant versions immobilized on HA-beads were incubated with 1µg GST-S6 and 5µCi P^32^ATP in a kinase reaction buffer containing 50mM Tris-Cl (pH 7.0), 10mM MgCl2, 0.5mM DTT, 50mM β-Glycero-phosphate, and 1mM ATP for 20 minutes at 37°C. Reaction was stopped by adding 6X loading buffer, run on a 12% SDS-PAGE gel. Proteins were transferred on PVDF membrane, Auto-radiographed, probed with different antibodies and analyzed using Odyssey infrared imager (LI-COR).Alternatively,S6 peptide was used as a substrate and activity determined by standard protocol .

**Phosphatase assays:** Active S6Kα1 WT as HA-conjugate with protein G-beads were washed thrice with PIPES buffer and then incubated with varying concentrations of Potato acid Phosphatase in PIPES buffer, containing 20mM PIPES (pH 4.8), 20mM KCl, 1mM DTT,1mM MgCl2 at 37°C for 30 minutes and then washed thrice with phosphatase inhibitor buffer containing 50mM Tris-Cl (pH 7.0), 10mM Sodium Fluoride , 1mM Sodium Ortho-Vanadate, 2mM Sodium Pyrophosphate deca-hydrate and 50mM β-Glycero-phosphate and once with kinase buffer. PP2A assay was done similarly in a buffer containing 20 mM MOPS, pH 7.5, 60 mM 2-ME, 0.1 M NaCl, and 0.1 mg/ml serum albumin) in a reaction volume of 50µl and reaction was stopped with 5ɳM okadaic acid.
